# Perspectives on push–pull chromophores derived from click-type [2 + 2] cycloaddition–retroelectrocyclization reactions of electron-rich alkynes and electron-deficient alkenes

**DOI:** 10.3762/bjoc.20.13

**Published:** 2024-01-22

**Authors:** Michio Yamada

**Affiliations:** 1 Department of Chemistry, Tokyo Gakugei University, Nukuikitamachi 4-1-1, Koganei, Tokyo 184-8501, Japan

**Keywords:** click chemistry, donor–acceptor conjugate, intramolecular charge transfer, photoluminescence, photoinduced electron transfer

## Abstract

Various push–pull chromophores can be synthesized in a single and atom-economical step through [2 + 2] cycloaddition–retroelectrocyclization (CA–RE) reactions involving diverse electron-rich alkynes and electron-deficient alkenes. In this review, a comprehensive investigation of the recent and noteworthy advancements in the research on push–pull chromophores prepared via the [2 + 2] CA–RE reaction is conducted. In particular, an overview of the physicochemical properties of the family of these compounds that have been investigated is provided to clarify their potential for future applications.

## Introduction

Push–pull chromophores, wherein both electron-donating and electron-accepting substituents are incorporated into a π-electron-conjugated system, exhibit exceptional optical and electronic properties. They hold significant promise for applications in diverse fields, particularly materials science (specifically in optoelectronics) [1–2]. The synthesis of push–pull chromophores is often achieved through click-type reactions between electron-rich alkynes and electron-deficient alkenes, which is a reliable and atom-economical method. Diverse chromophores can be obtained via this method, depending upon the choice of alkynes and alkenes. The pioneering work by Bruce et al. in 1981 revealed that the cleavage of tetracyanoethylene (TCNE) under mild conditions via its reaction with metal acetylides yields metal complexes featuring the tetracyanobuta-1,3-diene (TCBD) structural motif [3]. Subsequently, numerous researchers have explored the synthesis of various push–pull chromophores, primarily via reactions between alkynes and alkenes. Several comprehensive reviews of nonplanar push–pull chromophores have since enriched the scientific literature [4–6]. In 2018, Michinobu and Diederich provided an exemplary overview of click-type [2 + 2] cycloaddition (CA)–retroelectrocyclization (RE) reactions, offering means for preparing these coveted push–pull chromophores [7]. A review conducted in 2023 comprehensively explored the optoelectronic properties of TCBDs along with their photovoltaic applications [8]. The landscape of [2 + 2] CA–RE reactions for the preparation of push–pull chromophores is rapidly evolving and expanding. This evolution necessitates an update on the latest insights for materials science researchers. Furthermore, the diverse research advancements related to the distinctive physicochemical properties of push–pull chromophores remain fragmented. Thus, the primary objective of this review is to consolidate the developments in the research on push–pull chromophores prepared via [2 + 2] CA–RE reactions between electron-rich alkynes and electron-deficient alkenes across various domains of chemistry. This endeavor aims to delineate new directions for prospective applications of push–pull chromophores. Notably, in polymer chemistry, significant progress has been made in employing [2 + 2] CA–RE reactions for polymers as a valuable post-functionalization treatment [9–10]. However, this topic lies beyond the scope of this review.

## Review

### Click-type [2 + 2] CA–RE reactions

**Scheme 1:**
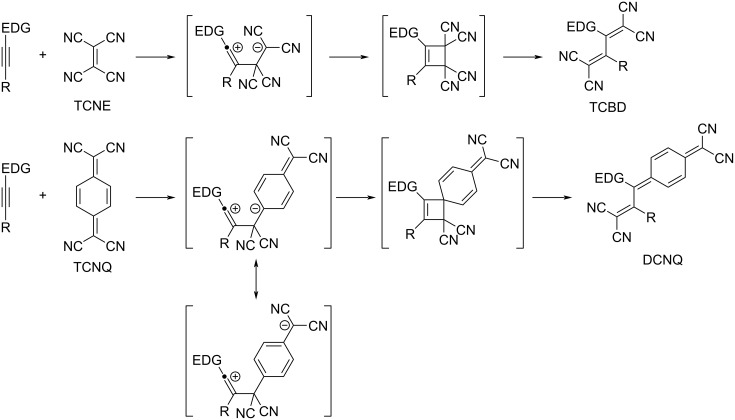
Pathway of the [2 + 2] CA–RE reaction of an electron-rich alkyne with TCNE or TCNQ. EDG = electron-donating group.

Click-type [2 + 2] CA–RE and associated reactions were comprehensively elucidated in a previous review [7]. Succinctly, the [2 + 2] CA reaction is postulated to be a stepwise process, whereas RE is postulated to be a concerted process. The [2 + 2] CA–RE sequence proceeds successively, as depicted in Scheme 1, where electron-donating groups are denoted as EDGs. During the [2 + 2] CA process, the nucleophilic attack by the terminal alkyne carbon of an electron-rich alkyne on an electron-deficient alkene, such as TCNE and 7,7,8,8-tetracyano-*p*-quinodimethane (TCNQ), results in the formation of a zwitterionic intermediate, wherein the negative charge evolves into a stabilized carbanion (dicyanomethide anion). Subsequently, the ring closure of the zwitterionic intermediate generates the corresponding cyclobutene intermediate. Finally, the ensuing RE process yields the corresponding TCBD derivatives. The resulting TCBDs and related products exhibit strong light absorption, resulting from the intramolecular charge transfer (ICT) in the visible region; they also exhibit a rich redox chemistry [11]. In the [2 + 2] CA–RE reaction of TCNQ with electron-rich alkynes, the alkyne terminal carbon executes a nucleophilic attack on the exocyclic carbon of the dicyanovinyl (DCV) group of TCNQ, affording dicyanoquinodimetanes (DCNQs) [12–13]. Intense ICT bands of TCBD and DCNQ are observed at around 450–470 nm and 680–710 nm, respectively. A study confirmed that with appropriate molecular design, the π-conjugation relationship between the donor and acceptor moieties in TCBDs and DCNQs can be retained despite their non-planarity [14]. Diederich et al. synthesized a plethora of push–pull chromophores by employing anilino groups as EDGs [4–5,7]. Notably, the *N*,*N*-dimethylanilino (DMA) moiety activates the reactivity of the neighboring alkyne moiety so strongly that the [2 + 2] CA–RE reactions with electron-deficient olefins proceed seamlessly [15–16] even when the terminus of the alkyne moiety is substituted by a cyano group [17]. In addition to anilino groups, a myriad of donor entities, including urea-substituted phenyl groups [18–19], carbazoles [20], phenothiazines [21–22], thiophenes [23–26], tetrathiafulvalenes (TTFs) [27], extended TTFs [28], ferrocenes [27,29–46], azulenes and their homologous compounds [32,47–58], boron dipyrromethenes (BODIPYs) [41,59–61], porphyrins [62–64], chlorophylls [65–66], triazenes [67–68], ynamides [69–71], arylynamines [72], indoles [73], and γ-pyranylidenes [74], have been identified as effective donor components for [2 + 2] CA–RE reactions. Concerning electron-deficient alkenes, 2,3,5,6-tetrafluoro-7,7,8,8-tetracyano-*p*-quinodimethane [75], DCV derivatives [76–80], tricyanovinyl derivatives [79–80], polyenic DCVs [81], *N*,*N’*-dicyanoquinone diimides [82], and 6,6-dicyanopentafulvenes (DCFs) [83–84] have been employed. Notably, the [2 + 2] CA–RE reaction of DMA*-*ethynyl-appended porphyrin with TCNQ is observed to occur on a metal surface (specifically Au(111)) under high-vacuum conditions, with successful visualization achieved through scanning tunneling microscopy [85]. For TCBDs bearing unsubstituted anilino (*p*-H_2_NC_6_H_4_–) groups, their conversion into the *p*-iodophenyl derivatives via the Sandmeyer reaction and subsequent post-functionalization via the Suzuki and Sonogashira coupling reactions are achieved [86]. In the reaction of bisanilino-end-capped buta-1,3-diynes with TCNE, simultaneous [2 + 2] CA–RE reactions proceed for each of the two alkyne moieties of the buta-1,3-diyne skeletons, forming octacyano[4]dendralene molecules corresponding to TCBD dimers [87–88]. A well-recognized potent electron acceptor, 2,3-dichloro-5,6-dicyano-1,4-benzoquinone, and its homologous compounds have been employed in chemical transformation reactions involving electron-rich alkynes. In particular, a [2 + 2] CA adduct was prepared through the [2 + 2] CA–RE reaction. Studies have shown that the thermal treatment of the [2 + 2] CA adduct leads to the formation of a spiro compound [89–94]. Ester-substituted, electron-deficient alkenes have also been employed in [2 + 2] CA–RE reactions involving electron-rich alkynes. Alkenes featuring either one or two ester substitutions exclusively catalyze [2 + 2] CA–RE reactions, yielding multicyanated ethenes [95]. Contrarily, alkenes bearing three or four ester substitutions partake in a [4 + 2]-type hetero-Diels–Alder (DA) reaction, yielding a third product, presumably through a [3 + 2] cycloaddition reaction, followed by rearrangement. The [2 + 2] CA–RE reactions involving cumulenes and TCNE also present noteworthy chemical transformation reactions [96–99]. However, the chemistry of cumulenes is not discussed in detail here; please refer to a previous review [7] for comprehensive insights.

**Scheme 2:**
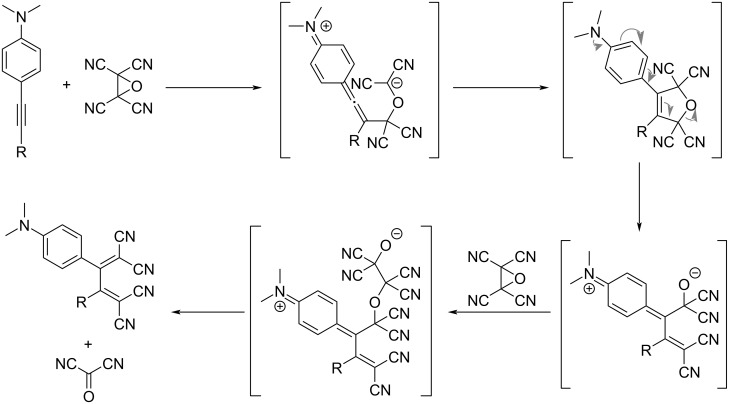
Reaction pathway for DMA-appended acetylene and TCNEO.

Upon replacing TCNE with tetracyanoethylene oxide (TCNEO), the [2 + 2] CA–RE reaction is extremely slow when applied to mono-substituted alkynes bearing an DMA group. Conversely, for bis-substituted alkynes, the reaction occurs readily at room temperature, yielding the corresponding TCBD products, as shown in Scheme 2. This reaction required the presence of two equivalents of TCNEO relative to the alkyne substrate for the generation of the TCBD products [100]. For the reaction, the formal [3 + 2] cycloaddition reaction is postulated to initiate through the initial nucleophilic attack of the alkyne carbon on the electrophilic TCNEO carbon, yielding a zwitterionic intermediate. Subsequently, following the production of an oxide ion through the ring-opening reaction of the 2,5-dihydrofuran ring, the oxide ion attacks another TCNEO molecule. This sequence culminates in the elimination of the tetracyanodioxetane moiety (either as dioxetane or carbonyl dicyanide molecules in the form of degraded fragments), affording the TCBD products.

**Scheme 3:**
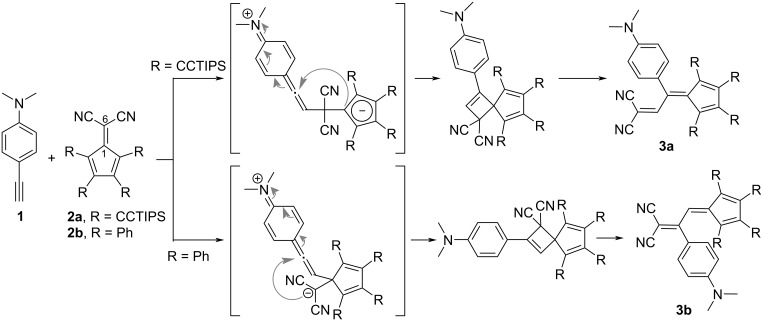
Pathway of the [2 + 2] CA–RE reaction between **1** and DCFs.

A distinguishing feature of the [2 + 2] CA–RE reaction involving electron-rich alkynes and DCFs is the alternation of regioselectivity, which is contingent upon the specific substituents incorporated onto the cyclopentadienyl moiety of the DCF molecule [83–84], as shown in Scheme 3. In the reaction between 4-ethynyl-*N*,*N*-dimethylaniline (**1**) and triisopropylsilylethynyl-substituted DCF **2a**, heating at 80 °C in acetonitrile selectively yields the corresponding adduct **3a** with 64% yield. In **3a**, the anilino group forms a covalent linkage, engaging in conjugation with the fulvene moiety. Conversely, the reaction of phenyl-substituted DCF **2b** with **1** under the same reaction conditions selectively affords the corresponding adduct **3b** with 64% yield. In **3b**, the anilino group is conjugated with the DCV group rather than the fulvene moiety. This variation in the products arises from the difference in the initial reaction step, in particular, whether the nucleophilic attack of the alkyne carbon in **1** occurs at C(6) or C(1) of the DCF framework. This differentiation can be attributed to the efficient electron conjugation between the alkyne and cyclopentadiene moieties.

**Scheme 4:**
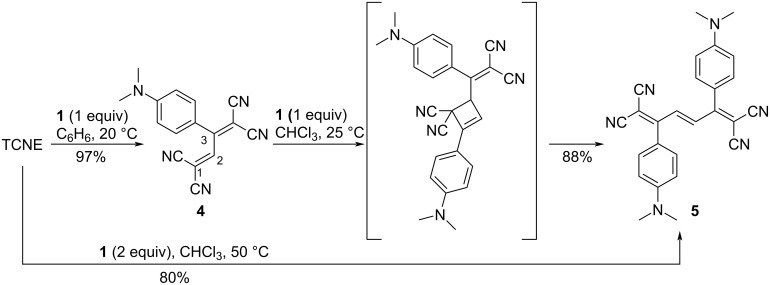
Sequential double [2 + 2] CA–RE reactions between **1** and TCNE.

**Scheme 5:**
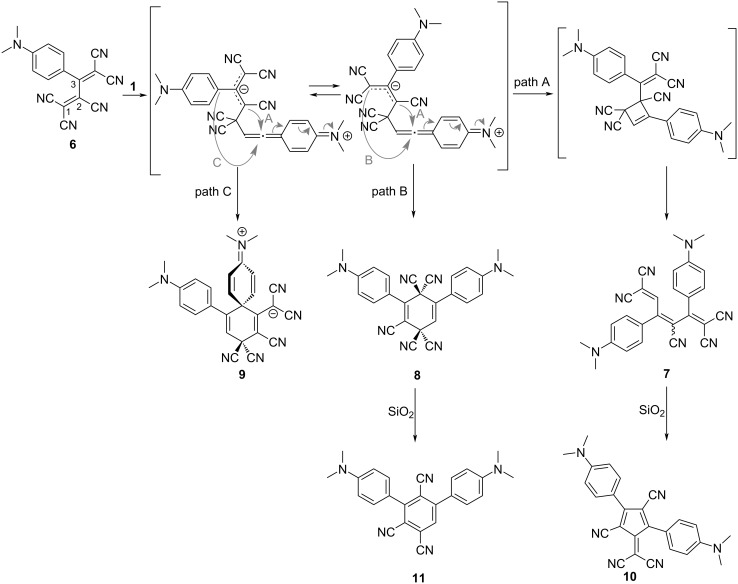
Divergent chemical transformation pathways of TCBD **6**.

TCBD **4**, obtained through the [2 + 2] CA–RE reaction, continues to function as an electron-accepting alkene, as shown in Scheme 4. Subsequent [2 + 2] CA–RE reactions involving electron-rich alkynes yield tetracyano-1,3,5-hexatrienes (TCHTs). These reactions occur seamlessly in a one-pot fashion. For instance, the reaction of one equivalent of TCNE with two equivalents of **1** in chloroform at 50 °C for 36 h yields the corresponding TCHT **5** with 80% yield [101]. In this reaction, the nucleophilic attack of the alkyne carbon of **1** occurs at the C(2) carbon of **4**. Moreover, when TCBD **6**, exhibiting enhanced electron-accepting capabilities, reacts with **1**, it displays a more intricate reaction profile compared with that of **4** (Scheme 5). In addition to pentacyano-1,3-5-hexatriene (PCHT) compound **7**, compounds **8** and **9** are generated [102]. This reaction is notably influenced by the different conditions, particularly the solvent employed. For instance, when toluene is used as the reaction solvent, a reaction at 100 °C for 15 h affords an *E*/*Z* mixture of **7** (*E*/*Z* ≈ 1:1) with 90% yield, **8** with 10% yield, and **9** with 0% yield. In contrast, a reaction at 25 °C for 15 h in *N*,*N*-dimethylformamide (DMF) affords **7** with 16% yield, **8** with 25% yield, and **9** with 46% yield. Furthermore, **7** and **8** are efficiently converted into **10** and **11**, respectively, with high yields (80% and 95%, respectively) via column chromatography employing silica gel in acetonitrile, thereby instigating further molecular transformation reactions. In these reactions, the nucleophilic attack of the alkyne carbon of **1** occurs at the C(1) carbon of **6**. When the formal [2 + 2] cycloaddition, as delineated in path A, occurs for the zwitterionic intermediate featuring the 1,1,3-tricyanoallyl anion obtained through the nucleophilic attack, subsequent RE affords **7**. Conversely, if the formal [4 + 2] cycloaddition occurs along the course elucidated in path B, the generation of **8** is expected. Similarly, when the formal [4 + 2] cycloaddition follows the course depicted in path C, the generation of **9** is envisaged. Among these compounds, **10** features a DCF structure and is amenable to further molecular transformations through a reaction with **1** [103].

**Scheme 6:**
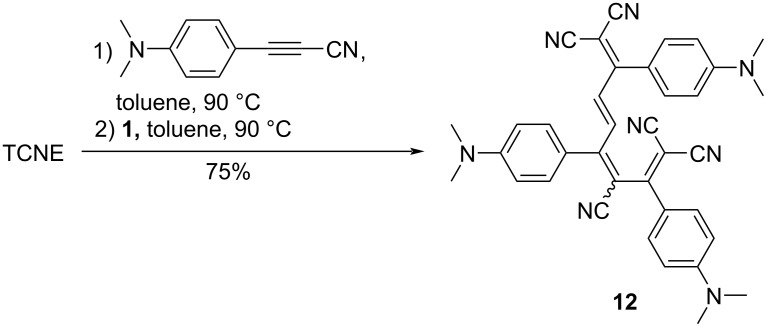
Synthesis of **12**.

Considering the structure of **7**, the possibility of a multistep [2 + 2] CA–RE reaction via a reaction with **1** becomes feasible. To execute such a multistep [2 + 2] CA–RE reaction, it becomes imperative to suppress the generation of **8** and **9**, as well as the conversion reaction of **7** into **10**. Therefore, it is important to use toluene as the solvent. Accordingly, pentacyanoocta-1,3,5,7-tetraene (PCOT) **12** was synthesized with 75% yield as a mixture of 3*Z*,5*E*/3*E*,5*E* isomers in the ratio of 71:29 via a one-pot reaction involving TCNE and one equivalent of 3-(4-(dimethylamino)phenyl)propionitrile in toluene at 90 °C for 24 h. Subsequently, one equivalent of **1** was added at 90 °C for 24 h (Scheme 6) [101]. While the separation of the isomers was not achieved, the structure of the 3*Z*,5*E* isomer was successfully characterized through single-crystal X-ray crystallography.

**Scheme 7:**
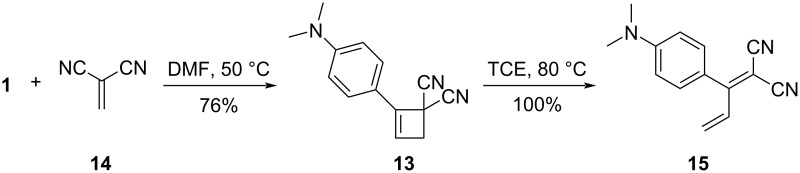
[2 + 2] CA–RE reaction of **1** with **14**. TCE = 1,1,2,2-tetrachloroethane.

A mechanistic investigation of the [2 + 2] CA–RE reactions involving DCV compounds was undertaken by Diederich et al. in 2010 [77]. Their investigation unveiled that the reaction between **1** and arylated DCV derivatives followed second-order kinetics, indicating a bimolecular process. Furthermore, their findings elucidated a compelling linear free-energy relationship between the rate constants and electronic characteristics of the *para*-substituents of the DCV electrophiles, implying a dipolar, zwitterionic mechanism. The researchers also performed theoretical calculations, unveiling that the ring-opening step presented a higher energy barrier compared with the ring-formation step, particularly when the DCV compounds lacked aryl substituents. Subsequently, they successfully isolated the corresponding cyclobutene intermediate **13** through the reaction of **1** with 1,1-dicyanoethene (**14**). They demonstrated that this intermediate was converted into the corresponding TCBD **15** upon further heating, as depicted in Scheme 7. The kinetics of the RE step conformed well to that of a first-order reaction. However, notably, they underscored that generalizing the elucidated reaction mechanism to other [2 + 2] CA–RE reactions involving TCNE and TCNQ as electrophiles might be difficult. They emphasized the significance of considering a pre-equilibrium state of the charge-transfer complexes between the alkynes and alkenes and mentioned that the positioning of the [2 + 2] CA or RE step as the rate-determining step may depend upon the structural attributes of the electrophiles and nucleophiles.

**Scheme 8:**
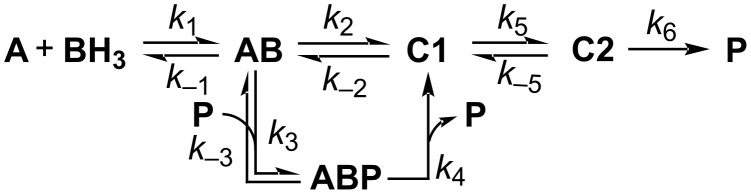
Autocatalytic model proposed by Nielsen et al.

In 2023, Nielsen et al. conducted an exhaustive kinetic analysis of the [2 + 2] CA–RE reaction involving 4-trimethylsilylethynylaniline and TCNE by leveraging ^1^H nuclear magnetic resonance (NMR) spectroscopy. Their investigation revealed that the product plays a pivotal role as an autocatalyst in the kinetics, as illustrated in Scheme 8 [104–106]. The traditional model posits that an alkyne (**A**) and TCNE (designated as **B** in Scheme 8) initially form a charge-transfer complex **AB**. Subsequently, this complex forms a zwitterion intermediate (**C1**), followed by the formation of a cyclobutene intermediate (**C2**), ultimately forming the product **P**. However, the researchers strongly advocated for an alternative pathway, which entails the formation of a complex between **AB** and **P**, from which **C1** is generated. Notably, this additional route significantly accelerates the overall reaction. The researchers reported that the conversion of the **ABP** complex into **C1** transpired at a markedly accelerated rate compared with the conversion of **AB** alone, with a kinetic constant ratio of *k*_4_/*k*_2_ = 62. Consequently, it is posited that **P** assumes the role of a template, directing the arrangement of reactants in a precise manner conducive to the generation of **C1**.

**Scheme 9:**
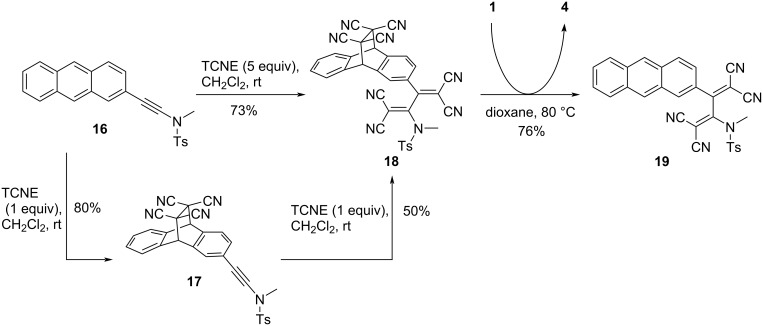
Synthesis of anthracene-embedded TCBD compound **19**.

Leveraging the irreversibility inherent in the [2 + 2] CA–RE reaction, **1** can be adeptly employed as a proficient trapping agent for TCNE. Anthracene-based ynamide **16** offers two potential reactive sites for TCNE, one residing at the anthracene moiety and other at the alkyne moiety, as shown in Scheme 9 [71]. As exemplified by Trolez et al., the introduction of one equivalent of TCNE to **16** at room temperature initiates a [4 + 2] cycloaddition reaction with the anthracene moiety, yielding the DA cycloadduct **17**. To achieve the conversion of the alkyne moiety of **16** into TCBD, the addition of five equivalents of TCNE to **16** or one equivalent of TCNE to **17** is required. This results in the formation of TCBD compound **18** bearing a derivatized anthracene moiety. Subsequently, to restore the anthracene structure of **18**, the retro-DA reaction must occur efficiently. Therefore, through the addition of **1**, followed by heating, the generated TCNE is effectively captured by **1**. This maneuver concurrently suppresses the progression of the DA reaction, leading to the efficient generation of the anthracenyl TCBD **19**.

**Scheme 10:**
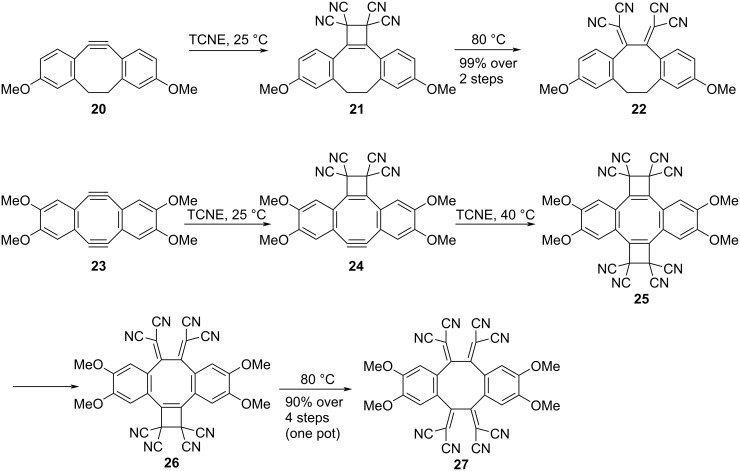
Sequence of the [2 + 2] CA–RE reaction between dibenzo-fused cyclooctyne or cyclooctadiyne and TCNE. All reactions were performed in TCE-*d*_2_.

Concerning [2 + 2] CA–RE reactions, studies have shown that alkynes exhibiting angular distortion exhibit high reactivity toward electron-deficient alkenes independent of the presence or absence of electron-donating substituents on the alkynes. The remarkable reactivity displayed by angle-strained alkynes makes them highly valuable tools in biorthogonal chemistry [107–109]. The reaction between dibenzo-fused cyclooctyne **20** and TCNE was observed to occur at room temperature, quantitatively yielding **22**, wherein the TCBD moiety was effectively integrated, as shown in Scheme 10 [110]. During this reaction, an intermediate **21** was successfully captured experimentally along with the occurrence of only the [2 + 2] CA reaction. Similarly, the reaction involving cyclooctadiyne (**23**) and TCNE progressed in a stepwise manner, ultimately affording **27** with 90% yield. The formation of intermediates **24**, **25**, and **26** was elucidated through ^1^H NMR analysis, of which **24** and **26** were successfully isolated and characterized. A comprehensive analysis of the [2 + 2] CA–RE reaction activation parameters via ^1^H NMR spectroscopy revealed that the rate-determining step in the transformation of **20** into **22** was the first-order RE step, which was primarily governed by enthalpy changes. Furthermore, it was observed that the ring strain significantly accelerated the second-order [2 + 2] CA step. The rate enhancement in the [2 + 2] CA step was approximately 3300 times that for an acyclic model compound, at 298 K. Regarding the conversion reaction from **23** to **27**, the rate-determining step was identified to be the final RE step: the conversion of **26** to **27**. Comparative analysis using a linear model compound revealed rate enhancements of 5500 times that for the initial [2 + 2] CA step and 80 times that for the subsequent [2 + 2] CA step at 298 K, which was attributable to the ring strain.

**Scheme 11:**
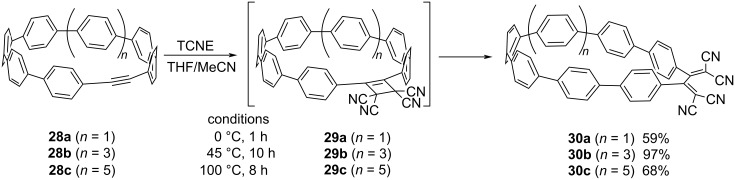
[2 + 2] CA–RE reaction between the CPP derivatives and TCNE. THF = tetrahydrofuran.

Jasti et al. synthesized an array of cycloparaphenylenes (CPPs) that incorporated alkyne moieties (**28a–c**) within their ring structures and investigated their reactions with TCNE (Scheme 11) [111]. In these reactions, CPP derivatives **30a–c**, featuring TCBD moieties, were obtained with 59–97% yields. The magnitude of the ring strain exerted a profound effect on the reactivity of the compounds. Notably, the largest macrocycle **28c** required heating to 100 °C to generate **30c**. Conversely, the transformation of **28b** proceeded at 45 °C, and the smallest macrocycle **28a** reacted with TCNE at 0 °C. Kinetic investigations revealed that the rate-determining step in the reaction involving **28a**–**c** with TCNE was the second-order [2 + 2] CA step, succeeded by the rapid RE step; the result is in contrast to those obtained for **20** and **23**. This divergence in the reactivity may be attributed to the pronounced steric repulsion between the DCV group and the aryl hydrogen, particularly discernible for **21** and **26** during the RE step. Conversely, for **29a**, it is reasonable to posit that the phenyl rings possess a degree of rotational freedom, thereby circumventing steric repulsions.

**Scheme 12:**
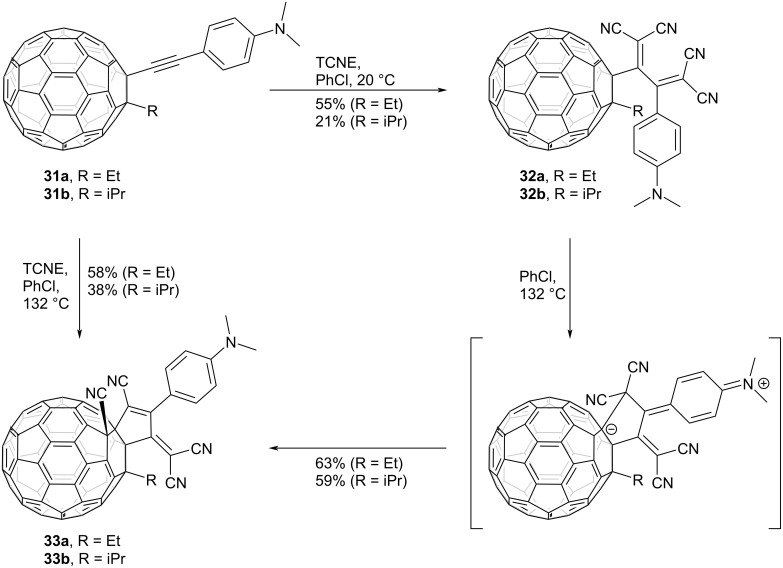
[2 + 2] CA–RE reaction between ethynylfullerenes **31** and TCNE and subsequent thermal rearrangement.

The steric congestion surrounding TCBD moieties can also induce further skeletal rearrangement. For instance, the spatial constraints proximal to the surface of fullerene derivatives lead to novel reactions arising from steric congestion. In a reaction involving ethynylfullerene derivatives featuring a DMA group (**31**) and TCNE, the conventional [2 + 2] CA–RE reaction was observed to transpire at 20 °C, yielding the corresponding TCBD derivatives **32**, as shown in Scheme 12. Conversely, upon refluxing in chlorobenzene, a rearrangement ensued, yielding tetrakis-substituted fullerene derivatives **33** [112]. During this rearrangement, it was postulated that a zwitterionic intermediate was formed from the nucleophilic attack of the carbon of the dicyanomethylidene adjacent to the DMA group on the fullerene sphere. This was followed by a nucleophilic attack of the fullerene carbanion on one of the cyano groups, ultimately yielding **33**. Computational calculations also suggested that the rearrangement was facilitated by the destabilization of the reactant due to steric interactions.

**Scheme 13:**
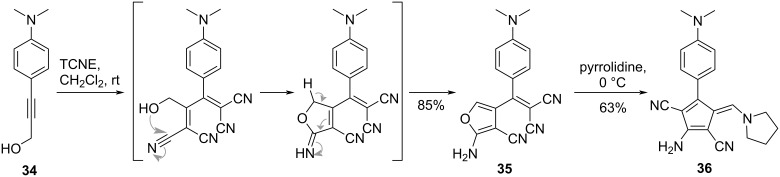
Pathway of the [2 + 2] CA–RE reaction between TCNE and **34**, followed by additional skeletal transformation.

Additional skeletal transformation reactions in TCBD structures, such as those shown in Scheme 12, have also been reported [113–116], which enable the construction of a wide array of push–pull chromophore structures. For instance, Shoji et al. reported that the reaction between 3(4-(*N*,*N*-dimethylanilino))prop-2-yn-1-ol (**34**) and TCNE in dichloromethane at room temperature yielded **35**, which was characterized by a furan skeleton (Scheme 13) [116]. This transformation occurred through the intermediate formation of the corresponding TCBD with a high yield of 85%. Furthermore, when **35** reacted with pyrrolidine at 0 °C, the pentafulvene derivative **36** was obtained with 63% yield via an additional ring-opening process. Similar skeletal transformation reactions were observed for azulene derivatives bearing similar substructures.

**Scheme 14:**
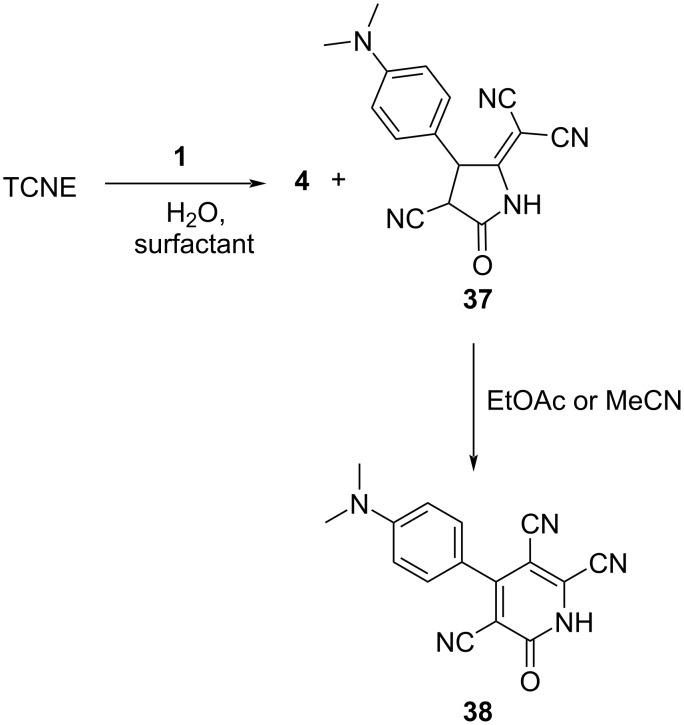
Synthesis scheme for heterocycle **38** from the reaction between TCNE and **1** in water and a surfactant.

The reaction of **1** with TCNE in an aqueous medium containing a surfactant results in the formation of a 6,6-dicyano-heteropentafulvene derivative **37** as an additional byproduct along with the typical TCBD adduct **4** (Scheme 14) [117]. The yields and formation ratios of **4** and **37** depend on the type and concentration of the surfactant employed. For instance, when the nonionic surfactant, Brij 30, was used as the reaction solvent at a concentration of 20 mM, exceeding the critical micelle concentration, **4** and **37** were obtained in a ratio of 53:47, with 64% yield. Notably, **37** exhibits instability in silica gel and polar solvents. In particular, when **37** is kept in ethyl acetate or acetonitrile for 8 h, **38** is obtained with 98% yield. Microscopic observations suggest that the vesicle structure, with water as the core, plays a pivotal role in the transformation of **4** into **37**. A similar reaction of TCBD with water was also reported by Bruce et al. [118].

**Scheme 15:**
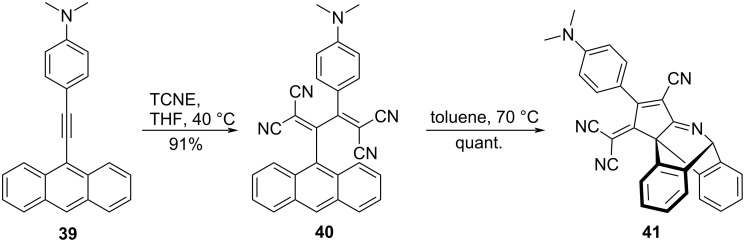
Synthesis scheme of the CDA product **41**.

The reaction of the anthracene derivative **39**, wherein a DMA–ethynyl group is introduced at the 9-position, with TCNE at 40 °C in THF yielded the corresponding TCBD **40** with 91% yield. Subsequent heating of **40** in toluene at 70 °C led to an intramolecular cyano-DA (CDA) reaction, which quantitatively afforded the CDA product **41** (Scheme 15) [119]. The quantitative CDA reaction progression was attributed to be due to the presence of multiple cyano groups on the skeleton, which increased the dienophile character of the cyano groups and stabilized the structure of the product. The thermal conversion of **40** to **41** was also accelerated by the addition of B(C_6_H_5_)_3_, which may be due to the enhancement of the dienophilic nature of the cyano group due to coordination with borane.

### Rotaxane synthesis

**Scheme 16:**
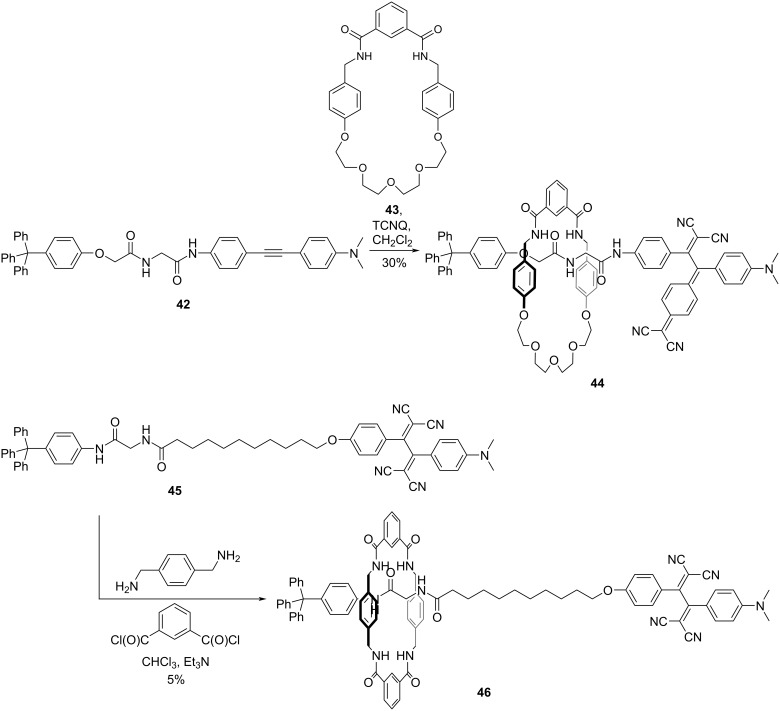
Synthesis of rotaxanes **44** and **46** via the [2 + 2] CA–RE reaction.

Push–pull chromophores with nonplanar configurations have been reported as effective stoppering motifs for rotaxane synthesis. A rational approach toward rotaxane synthesis involves affixing stoppers at both termini substituents and threading a molecular thread through a macrocycle. In this threading–stoppering strategy, a mild yet high-yield reaction is required for the stoppering process. The [2 + 2] CA–RE reaction, which yields push–pull chromophores, is the preferred method for stoppering. Accordingly, Li et al. synthesized a rotaxane **44** terminated using push–pull chromophores by incorporating concise peptide and DCNQ moieties and employing the threading–stoppering method (Scheme 16). In **44**, the rod moiety and macrocycle feature short peptide substructures. Consequently, the macrocycle was tethered to the peptide segment through amide–amide hydrogen bonding, and **44** was obtained with 30% yield. The generation of **44** was accompanied by the formation of the corresponding free thread with 62% yield, resulting from the reaction between the thread precursor **42** and macrocycle **43**, followed by an additional reaction of the alkyne moiety with TCNQ [120]. Rotaxane **46** terminated using push–pull chromophores, which exhibited a solvent-driven molecular shuttling phenomenon, was synthesized from the thread precursor **45** using a clipping approach; however, the yield for the ring-formation process was modest at 5% (see Scheme 16) [121–122]. The macrocycle in **46** was observed to engage with the peptide segment in low-polarity solvents. In contrast, ^1^H NMR analysis suggested that the macrocycle disengaged from the peptide segment and relocated along the alkyl chain in dimethyl sulfoxide. Furthermore, the solvent-driven shuttling motion of the macrocycle was observed to exert a discernible effect on the aggregation of the amphiphilic molecular system. In particular, changing the solvent led to the generation of interlaced nanofibers, perforated capsules, and wormlike nanoparticles.

**Scheme 17:**
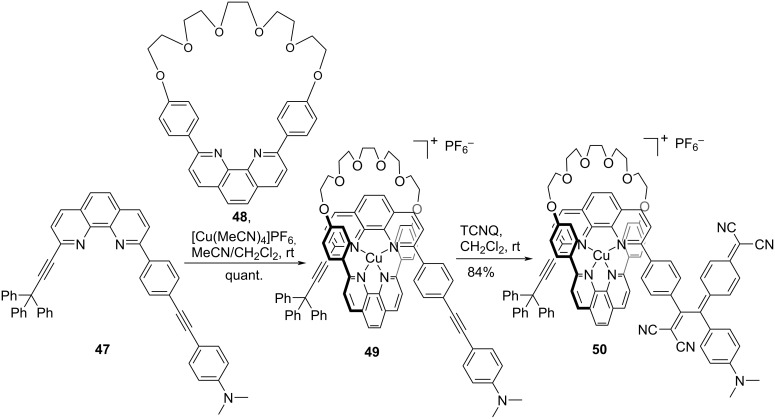
Synthesis of a Cu^I^ bisphenanthroline-based rotaxane **50**.

In rotaxanes, the utilization of metal–ligand bonding involving Cu^I^ is a common strategy for immobilizing a thread moiety within a macrocycle. However, the efficacy of such a bonding is compromised when catalysts are used in stoppering reactions, e.g., the copper-catalyzed azide–alkyne cycloaddition reaction. Consequently, a [2 + 2] CA–RE reaction that can yield push–pull chromophores without the use of a catalyst is exceedingly valuable as a stoppering method, even for systems featuring metal–ligand bonding. Accordingly, Diederich et al. demonstrated the synthesis of a Cu^I^ bis-phenanthroline-based rotaxane **50** by employing the [2 + 2] CA–RE reaction, as shown in Scheme 17 [123]. The initial threading reaction involving the rod molecule **47** and macrocycle **48**, constructed using [Cu(MeCN)_4_]PF_6_, resulted in the formation of the pseudorotaxane **49**. The ensuing [2 + 2] CA–RE reaction of **49** with TCNQ in dichloromethane at room temperature afforded **50** with an impressive yield of 84%.

### Chiroptical properties

**Figure 1:**
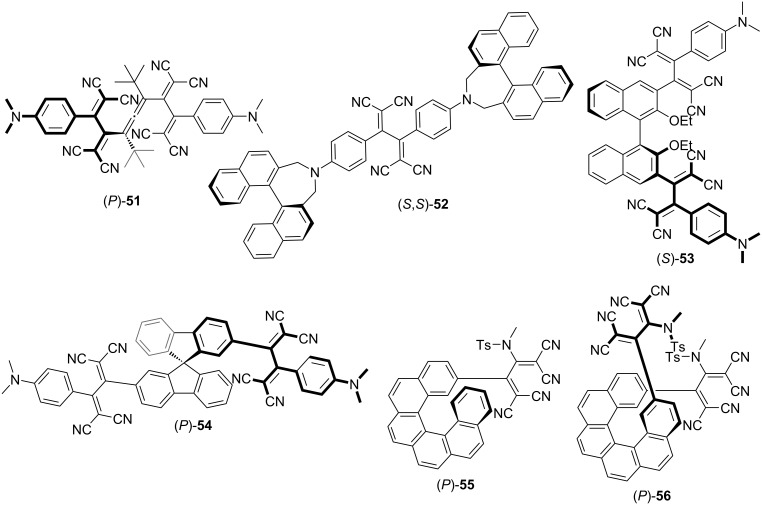
Structures of the chiral push–pull chromophores **51**–**56**.

Push–pull chromophores synthesized through the [2 + 2] CA–RE reaction can, in certain cases, exhibit distinct chiroptical properties, attributable to their nonplanar geometry and steric congestion. In previous studies, chiral induction in TCBD structures was accomplished by introducing chiral allene (**51**) [124–125] or binaphthyl (**52** and **53**) [126–127] moieties, as shown in Figure 1. These molecules exhibited Cotton effects related to ICT absorptions, and chiral induction in TCBD moieties resulted from optically active constituents. Compound **53**, due to its elongated rigid structure, holds potential for use as a chiral dopant in nematic liquid crystals (LCs); however, the helical twisting powers of **53** within nematic LCs are limited. Recently, Alonso-Gómez et al. reported the synthesis and optical resolutions of spirobifluorenes featuring two TCBD units located at the 2,2’-positions, designated as **54** [128]. Furthermore, Autschbach et al. prepared chiral carbo[6]helicene–TCBD derivatives **55** and **56** from enantiopure precursors [129]. For such intricate molecular systems, the presence of multiple chromophore units induces complexity in the interpretation of the resulting circular dichroism (CD) spectra because of the overlap of several exciton couplets. Thus, exciton coupling CD signals were not discernible for **51**–**55**. Further, regarding **56**, the exciton coupling CD signal in the ICT region may not be apparent at a glance. Nevertheless, comprehensive computational analyses employing the matrix method suggest that the intense long-wavelength CD signal observed for **56** is due to the coupling of individual helicene-to-TCBD electric-transition dipole moments.

**Figure 2:**
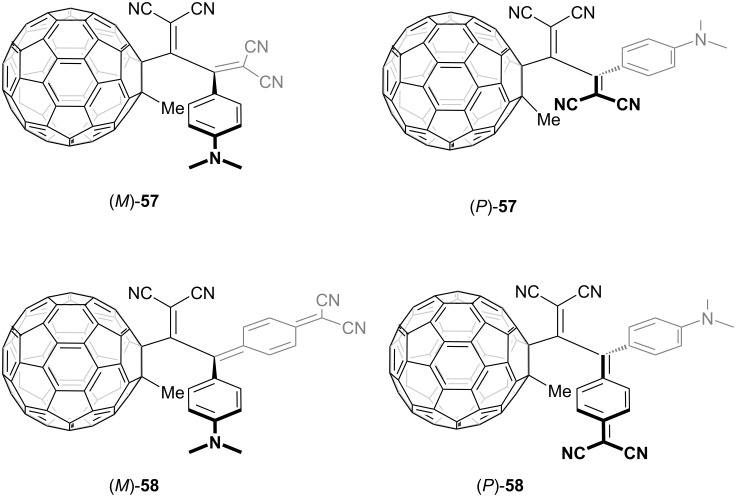
Structures of the axially chiral TCBD **57** and DCNQ **58** bearing a C_60_ core.

The emergence of axial chirality in TCBDs and DCNQs and their optical resolutions were first realized in 2010 through their conjugation with methylated fullerenes, as shown in Figure 2 [130]. The optical resolution was realized using a chiral high-performance liquid chromatography (HPLC) system equipped with an (*S*,*S*)-*WHELK*-*O*1 column. The absolute configurations of the axially chiral TCBD and DCNQ derivatives were ascertained by a comparative analysis of the experimental CD spectra against the spectra derived from time-dependent density functional theory (TD-DFT) calculations. The axial chirality was stabilized by the steric congestion on the surface of fullerene, with its endurance contingent upon the bulkiness of the substituent incorporated onto the fullerene core. This observation was supported by the absence of axial chirality in molecules where an additional alkyne moiety is intercalated between the C_60_ core and TCBD or DCNQ unit or where a methyl group on the C_60_ surface is replaced by a hydrogen atom. The C_60_–TCBD conjugate **57** and C_60_–DCNQ conjugate **58** exhibited substantial optical rotations at 589 nm, presenting +770 for (*P*)-**57** and +5,800 for (*P*)-**58**. In concordance, robust Cotton effects related to ICT absorption in the visible region were evident in their CD spectra. Using the Arrhenius and Eyring formalisms, the kinetic parameters for the thermal racemization of **57** and **58** were obtained. The activation free enthalpy (Δ*G*^‡^) for the racemization of **57** (Δ*G*^‡^_298 K_ = 24.8 kcal mol^−1^) was ≈1.5 kcal mol^−1^ larger than that for **58** (Δ*G*^‡^_298 K_ = 23.3 kcal mol^−1^), indicating that the cylohexa-2,5-diene-1,4-diylidene moiety is more flexible and its distortion by out-of-plane bending is energetically less favored compared to the DCV moiety.

**Figure 3:**
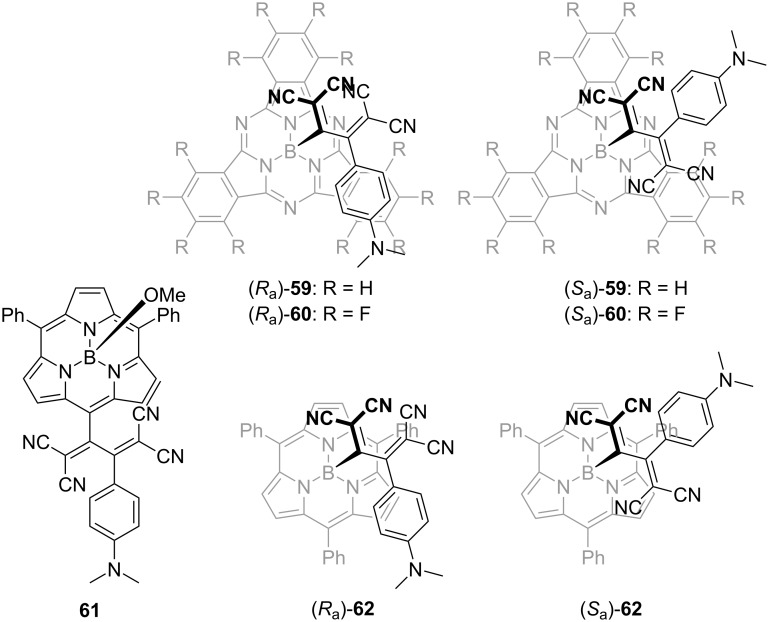
Structures of the axially chiral SubPc–TCBD–aniline conjugates **59** and **60** and the subporphyrin–TCBD–aniline conjugates **61** and **62**.

Guldi et al. reported that the atropisomerism in the TCBD structure was observed when the TCBD moiety was incorporated at the axial position of the subphthalocyanine (SubPc) core (Figure 3) [131]. Axially chiral SubPc–TCBD–aniline conjugates **59** and **60** were characterized via optical-resolution analysis through chiral HPLC using a Chiralpak IC column. The researchers unequivocally determined the absolute configurations of these atropisomers through the X-ray crystallographic analyses of (*S*_a_)-**59** and (*R*_a_)-**60**. Surprisingly, heating the enantiomers of **69** and **60** at 80 °C for 3 h resulted in negligible thermally induced racemization. Meanwhile, in O_2_-free toluene solution under illumination, the racemization was observed at 80 °C. Torres et al. performed detailed theoretical calculations and showed that the racemization observed in **59** and **60** is caused by triplet-state photogeneration, which leads to the rotation around the sterically hindered buta-1,3-diene chiral axis [132]. In fact, the estimated energy barrier of ≈37 kcal mol^−1^ is reduced to ≈15 kcal mol^−1^ upon the electronic excitation of **59** and **60** to the T_1_ state. It is reasonable to consider that the phototriggered racemization is inhibited in the presence of O_2_ because O_2_ acts as a triplet-state scavenger. Osuka et al. synthesized two types of subporphyrin derivatives **61** and **62**, wherein the subporphyrin skeleton was functionalized at its *meso* or axial position with the TCBD moiety (Figure 3). Although the optical resolution of **61** failed, they succeeded in achieving the optical resolution of **62** via chiral HPLC using a Chiralpak IC column. In **62**, unlike **59** and **60**, thermally induced racemization was observed and kinetic parameters were obtained (Δ*G*^‡^_298 K_ = 25.4 kcal mol^−1^), which were similar to those reported for **57** and **58** [133].

### Photoluminescence properties

**Figure 4:**
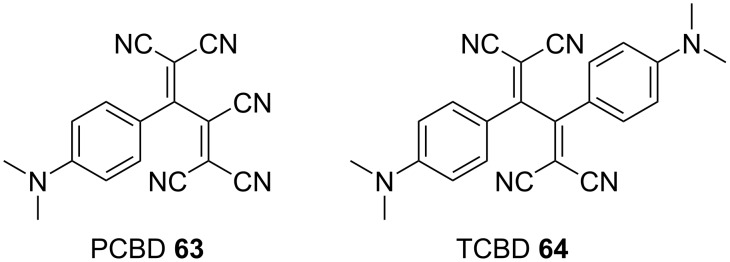
Structures of **63** and the TCBD **64**.

Most anilino-substituted TCBD molecules exhibit negligible fluorescence in chloroform, while only a few exhibit weak fluorescence in hexane [15]. These nonluminescence characteristics are attributable to the low-energy state of the twisted ICT (TICT) state, which is capable of deactivating the ground state through accessible conical intersections (CIs) [15,134–136]. In particular, Diederich et al. conducted a theoretical investigation on the photophysical properties of 1,1,2,4,4-pentacyanobuta-1,3-diene (PCBD) (**63**) [134]. Compound **63** and the TCBD **64** demonstrated no discernible luminescence at room temperature and 77 K (Figure 4). Transient absorption spectral measurements of **63** in toluene revealed that the lowest singlet excited state (S_1_) decays mono-exponentially to the ground state (S_0_) within approximately 1 ps, suggesting the existence of an accessible CI between S_1_ and S_0_. In the S_1_ minimum conformation of **63**, the PCBD moiety adopted a planar orientation perpendicular to that of the aniline moiety. The calculations indicated the presence of accessible S_1_/S_0_ CIs. In the lowest-energy S_1_/S_0_ CI geometry, the aniline moiety exhibited a pronounced quinoidal character and the carbon atom directly linked to the butadiene moiety exhibited a conspicuous radical nature. Concurrently, the excited electron was extensively delocalized over the entire pentacyanobutadiene moiety. The researchers postulated the following description for the photophysical properties of **64**: a nonradiative deactivation process occurred via a CI, which was similar to that observed in the case of **63**.

**Figure 5:**
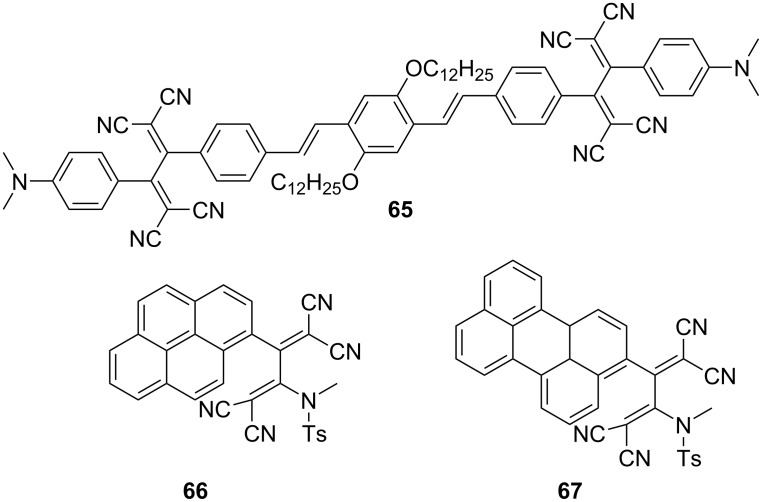
Structures of the fluorophore-containing TCBDs **65**–**67**.

Notably, a rigid molecular environment, which induces constraints on molecular motions, serves to mitigate nonradiative losses in push–pull chromophores. Li et al. synthesized an oligo(*p*-phenylenevinylene)-based TCBD derivative denoted as **65**, which is noteworthy for its lack of photoluminescence in solution as well as contradictory propensity to form luminescent nanostructure suspensions in hexane (Figure 5) [137]. It was posited that **65** features hollow vesicles, with vesicle fusion releasing curvature energy, leading to a thermodynamically more stable tubular structure. Such aggregation-induced-emission (AIE)-active aggregates exhibit emissive behavior in hexane, with an emission peak at 679 nm and a shoulder at 717 nm along with a fluorescence quantum yield of 8.5%. This luminescence phenomenon can be aptly considered to be AIE, considering the pronounced constraints imposed on molecular rotation in the aggregate state, including rotation around the central single bond of the TCBD moiety.

Regarding the anthracene-embedded TCBD **19** in dichloromethane, no emission signal was observed. However, in the powdered form or sparsely distributed rigid matrices, a broad emission spectrum was observed. In particular, the powders of **19** exhibited photoluminescence in the near-infrared (NIR) region centered at 865 nm, with a long tail extending up to 1,550 nm. This enhancement in photoluminescence can be attributed to the restriction of molecular motion, resulting in the inhibition of nonradiative processes. Analogously, the TCBDs **66** and **67**, consisting of pyrene and perylene moieties, respectively, have been reported to exhibit luminescence in the NIR region in their powdered forms, with the maxima at 810 nm and 890 nm for **66** and **67**, respectively, despite their nonemissive behavior in dichloromethane [138].

Compounds **55** and **56** remain devoid of emissive characteristics even when in a solution or a solid state [129]. When compound **50** was characterized using a standard steady-state fluorimeter, no emission signals were observed across the visible and NIR regions extending up to 1,600 nm, both in a room-temperature solution (dichloromethane) and as a frozen matrix at 77 K [123]. This is in contrast with the typical homoleptic phenanthroline-based CuI complexes renowned for their emissions from a triplet metal-to-ligand charge transfer excited state. The absence of luminescence may be attributed to the presence of competitive processes, such as energy or electron transfer.

**Figure 6:**
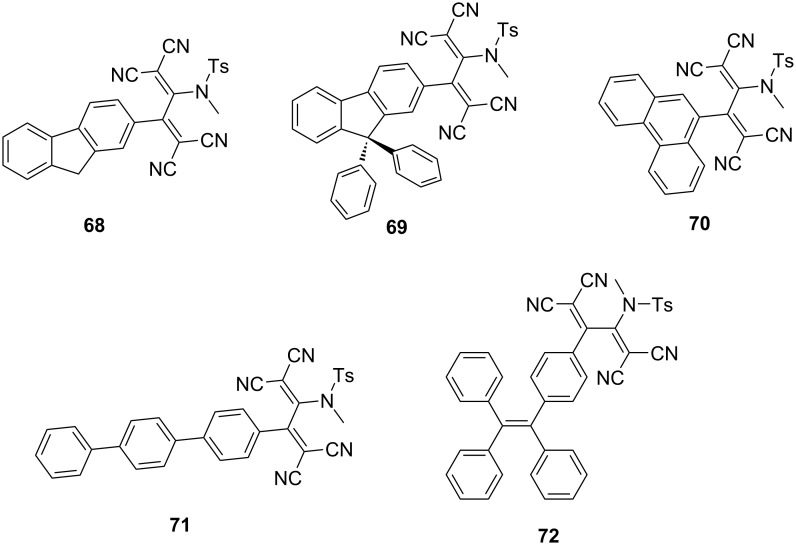
Structures of the fluorophore-containing TCBDs **68**–**72**.

Trolez et al. investigated the photoluminescence properties of various fluorophore-containing TCBDs synthesized via reactions between ynamides and TCNE [139]. The study revealed that numerous fluorenyl derivatives and their phenanthrenyl and terphenyl counterparts exhibited noteworthy emission behavior in solid form and in solutions (Figure 6). For instance, the fluorenyl derivatives **68** and **69**, phenanthrenyl derivative **70**, and terphenyl derivative **71** exhibited fluorescence characterized by emission peaks at 596, 595, 639, and 594 nm, with fluorescence quantum yields of 6.1%, 7.5%, 1.6%, and 7.8%, respectively, when dissolved in cyclohexane. Notably, the photoluminescence characteristics of these compounds depend on the solvent polarities, as evidenced by the observed decrease in the fluorescence quantum yields in toluene. This phenomenon aligns with the notion that the TICT states experience stabilization with increasing solvent polarity. Conversely, in contrast to **68**–**70**, and **71**, a TCBD derivative **72** incorporating a tetraphenylethylene (TPE) moiety exhibited no emission in toluene, possibly due to the presence of intramolecular motion within the propeller core of TPE, which promoted nonradiative decay [140].

**Figure 7:**
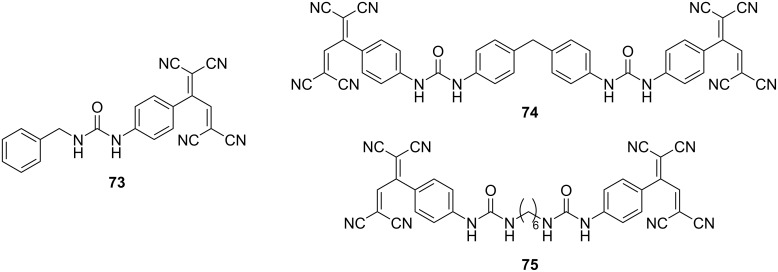
Structures of the urea-containing TCBDs **73**–**75**.

A subgroup of compounds that exhibit exceptional luminescence in solution includes **73**, **74**, and **75**, each containing urea-substituted phenyl groups (Figure 7) [18–19]. When excited at 380 nm, these compounds show broad emission spectra encompassing the entire visible region, featuring two peaks centered at 430–472 and 633 nm. The fluorescence quantum yields estimated for **73**, **74**, and **75** in acetonitrile are 3.4%, 3.3%, and 4.3%, respectively. Notably, in the case of **74** and **75**, the intensity of the long-wavelength emission increases when they are excited at 420 nm, resulting in white-light emission. The underlying mechanism governing these luminescence properties remains unknown. It has been established that **75** does not exhibit luminescence in its solid-state form, which is attributed to the quenching effects arising from hydrogen bonding and π–π stacking interactions. In contrast, when **75** is incorporated into a nanocomposite with polystyrene serving as the matrix, luminescent properties are observed [141].

### Photoinduced intramolecular energy and electron transfer

**Figure 8:**
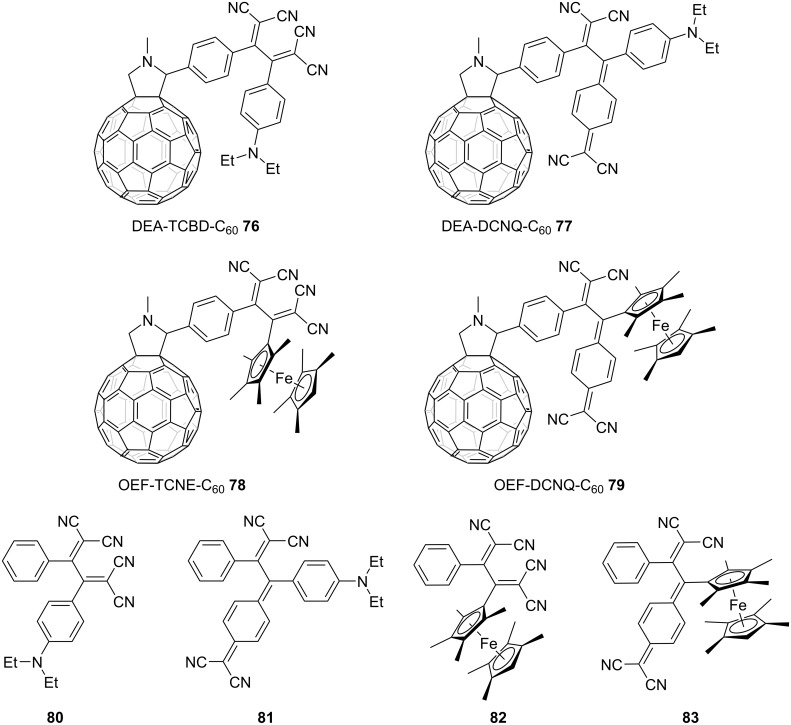
Structures of the fullerene–TCBD and DCNQ conjugates **76**–**79** and their reference compounds **80–83**.

Exploiting the electron-accepting property of TCBD, donor–acceptor conjugated systems have been systematically developed by coupling the TCBD motif with an electron donor, resulting in the experimental observation of photoinduced electron- and energy-transfer events. In 2014, comprehensive investigations on the photophysical properties of conjugates featuring diethylamino groups and TCBD or DCNQ structures covalently linked to C_60_ (**76** and **77**) and conjugates incorporating octamethylferrocenyl (OEF) groups and TCBD or DCNQ structures bonded to C_60_ (**78** and **79**) in addition to their respective reference compounds (**80**–**83**) were reported (Figure 8) [142]. In these conjugates, the push–pull chromophores and the C_60_ unit were effectively spatially isolated from each other – a feat achieved through the strategic incorporation of a pyrrolidine ring as the connecting bridge. Thorough examinations via steady-state fluorescence spectroscopy in toluene unequivocally demonstrated that compounds **77**–**83** exhibited negligible emissions upon excitation in the charge-transfer (CT) band. In contrast, compound **76** emitted radiations corresponding to fluorescence exhibited by fullerenes, with a peak wavelength of 708 nm, upon CT-band excitation. This observation confirms the plausible energy transfer from the local charge-separated (CS) state of the push–pull chromophore (namely *N*,*N*-diethylanilino (DEA)^•+^–TCBD^•−^) to the singlet excited state of C_60_ (^1^C_60_*). In transient absorption spectral measurements conducted via the femtosecond laser-flash photolysis of compound **80** in toluene, the occurrence of CT-band bleaching along with increased absorption in the 500–700 nm spectral range and a net decrease in absorption within the 850–1,200 nm region has been observed upon excitation at 420 nm, which is proximate to the CT band. For compound **76**, in addition to the CT-band bleaching and increased absorption within the 500–700 nm range, an increase in the absorption intensity at 1,021 nm, corresponding to the characteristics of the C_60_ radical anion, was observed initially (20 ps), indicating the formation of the DEA^•+^–C_60_^•−^ CS state. Furthermore, an increase in the absorption intensity at 700 nm, corresponding to the triplet excited state of C_60_ (^3^C_60_*), was observed along with the decay of the CS state with a lifetime of 1,380 ps. Conversely, for compound **77**, excitation at 387 nm was required for the formation of the CS state and an increase in the absorption intensity at 890 nm, indicating the emergence of ^1^C_60_* state with a lifetime of 115 ps, was accompanied by a subsequent increase in the absorption intensity at 1,021 nm, corresponding to the formation of the C_60_ radical anion. The lifetimes of the charge separation and charge recombination events were determined to be 2 and 165 ps, respectively, via multiwavelength analyses. The excitation at 640 nm, a wavelength proximate to the CT band, failed to induce any discernible electron or energy transfer from the DEA moiety to the C_60_ core. When compound **77** underwent photoexcitation at 640 nm, the CT band exhibited bleaching along with an increase in the absorption intensity at 523 nm and a decrease in the NIR region, similar to the trend observed for **80**. Notably, no other distinctive changes were observed. The photoexcitation of **78** at 420 nm caused CT band excitation, leading to charge separation. This was exemplified by the emergence of a maximum absorption peak at 1,024 nm, indicating the one-electron reduced form of C_60_. Although the observation of the oxidation process of the ferrocene unit was obstructed by the more substantial absorption changes associated with fullerene reduction, the lifetimes of the formation and decay of the CS state, OEF^•+^–C_60_^•−^, were determined to be 13 and 82 ps, respectively. For **79**, the excitation of C_60_ at 387 nm was essential for the development of the CS state, similar to that observed for **77**. In this case, the lifetimes of the charge separation and recombination events were determined to be 2 and 58 ps, respectively. The observed longer lifetimes attributed to DEA substitution could be due to the larger distance and the Marcus inverted region [143] character, compared with the results obtained for OEF substitution.

**Figure 9:**
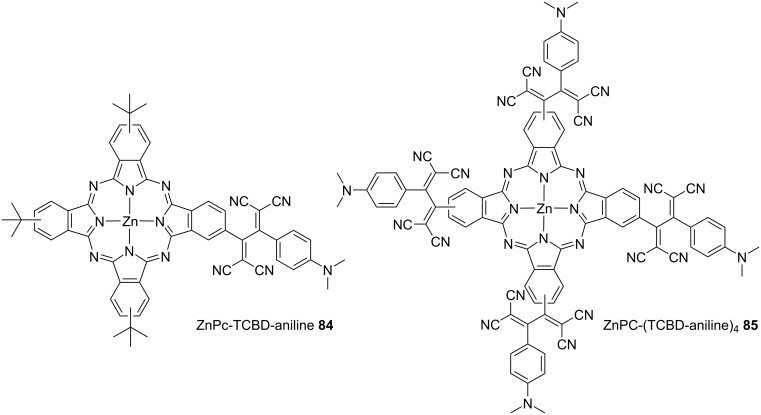
Structures of the ZnPc–TCBD–aniline conjugates **84** and **85**.

Guldi et al. synthesized zinc phthalocyanine (ZnP_C_) covalently linked with anilino-TCBD molecules, denoted as **84** and **85**, and investigated their optical properties (Figure 9). They observed robust low-energy ICT absorption peaks at 752/753 nm, along with the distinctive high-energy ICT absorption peaks at 470 nm, which are characteristic of anilino-TCBD. Furthermore, they identified B-band (355/361 nm) and Q-band (660/685 nm) transitions, which are hallmarks of phthalocyanines in benzonitrile, and elicit panchromatic optical absorption properties [144]. In **84** and **85**, the two DCV structures comprising TCBD are primarily contorted and exhibit limited conjugation. Consequently, these two structures could independently contribute to the emergence of two distinct and robust ICT bands in close proximity to those of their neighboring electron-rich ZnPc and DMA groups. Notably, in the transient absorption spectra of **84**, acquired upon excitation at 775 nm (an excitation wavelength corresponding to the low-energy CT absorption), the direct formation of a CS state, characterized as ZnPc^•+^–TCBD^•−^, can be observed.

SubPc–TCBD–aniline conjugates **59** and **60** exhibited distinct physicochemical properties, depending on whether the peripheral substituents introduced into the SubPcs consisted of hydrogen (as in **59**) or fluorine (as in **60**) atoms [131]. For **59**, the emergence of a metastable radical ion-pair state (H_12_SubPc^•+^–TCBD^•−^–aniline) was realized after the generation of the H_12_SubPc singlet excited state via 550 nm and 458 nm excitations, corresponding to the Q-band of H_12_SubPc and the ICT band, respectively. This indicates that similar ultrafast charge-separation and charge-recombination dynamics will also be observed in toluene. For **60**, the emergence of new maxima at 500, 660, and 742 nm and minima at 460 nm accompanied the decay of the excited singlet state of F_12_SubPc, which was first generated by 550 nm excitation in toluene. The transients at 500 and 742 nm were considered to result from the F_12_SubPc^•−^ radical anion, that at 660 nm was considered to result from the TCBD^•−^ radical anion, and the minimum at 460 nm corresponded to the bleaching of the ICT absorption band. This suggests that exciplexes ([(F_12_SubPc–TCBD)^δ−^–aniline^δ+^]*) are generated where the negative charge is delocalized over F_12_SubPc and TCBD. The radiative exciplex formation was also confirmed by time-resolved fluorescence measurements. In fact, in toluene, **60** showed maximum emission at 675 nm and a shoulder emission at 612 nm. When the solvent was changed from toluene to benzonitrile and the polarity of the solvent increased, the emission at 612 nm remained almost unchanged, while the emission at 675 nm significantly quenched with a red shift of 35 nm. In the transient absorption spectra of **60** in benzonitrile, the formation of an exciplex was not observed and it was considered that the CS state ((F_12_SubPc–TCBD)^•−^–aniline^•+^) was formed from the singlet excited state of F_12_SubPc before returning to the singlet ground state. The aforementioned phenomenon, characterized by the switch in the exciplex or the CS-state formation for **60** depending on the solvent, was also observed for excitation at 458 nm corresponding to the ICT band.

**Figure 10:**
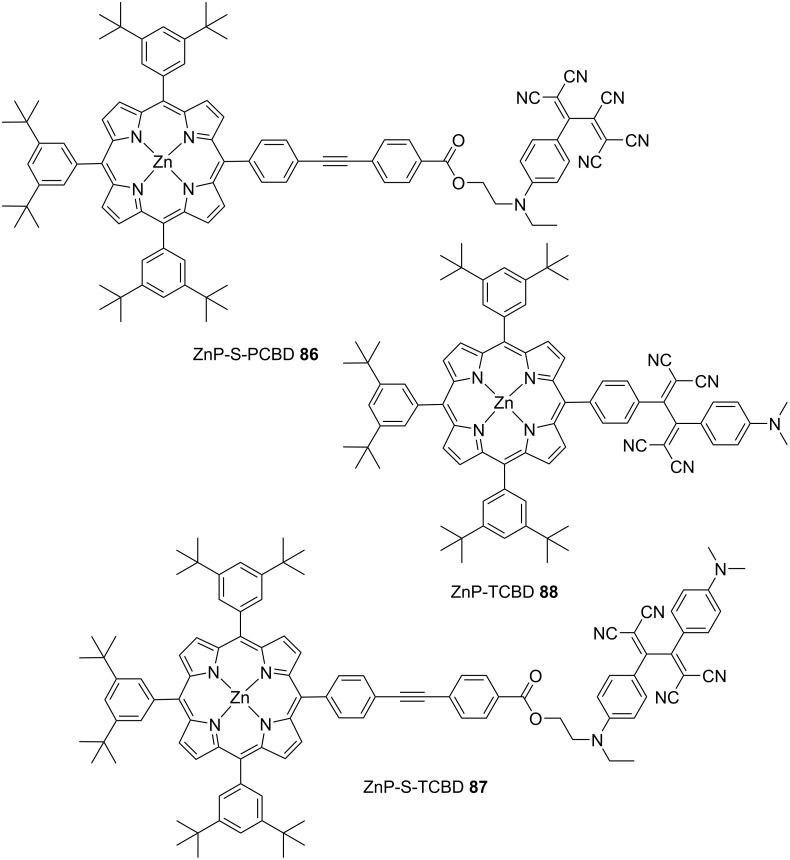
Structures of the ZnP–PCBD and TCBD conjugates **86**–**88**.

A series of multicomponent systems consisting of anilino-substituted TCBD or PCBD coupled with zinc porphyrins (ZnPs) were synthesized, and their photophysical properties were investigated by Diederich et al. [145]. The representative molecules among these are shown in Figure 10. In **86**, wherein ZnP and anilino-PCBD are linked using a spacer, the excitation of the porphyrin chromophore (at 560 nm, corresponding to the Q-band) generated an excited singlet state of ZnP (^1^ZnP*) with a lifetime of 71 ps in toluene. Meanwhile, the absorption at 640 nm, corresponding to the ZnP radical cation, was not observed. The results obtained from Rehm–Weller’s equation [146] suggested that photoinduced electron transfer was thermodynamically permitted in **86**. However, ultrafast energy transfer from ^1^ZnP* to the PCBD moiety was considered to have occurred. In **87**, the energy transfer from ^1^ZnP* to the TCBD moiety was not thermodynamically allowed. Conversely, the CS state (ZnP^•+^-S-TCBD^•−^) was estimated to be approximately isoenergetic. The lifetime of ^1^ZnP* was estimated to be 1,080 ps. However, the emergence of the weak absorption band of the ZnP radical cation at 640 nm was hindered by the overwhelming absorption intensity of the residual porphyrin. The transient absorption spectra of **87** were obtained in benzonitrile solvent, which was expected to stabilize the CS state. Absorption corresponding to the ZnP radical cation was clearly observed with a lifetime of 2.3 μs. The occurrence of such a long-living CS state can be rationally associated with the Marcus-inverted-region [143] behavior of the charge-recombination process. For **88**, which has no spacer between ZnP and TCBD, as opposed to the case for **87**, the excitation of the porphyrin chromophore in toluene led to the formation of ^1^ZnP* with a lifetime of 6.6 ps. This was followed by the emergence of the CS state and subsequent charge recombination within 20 ps. Thus, the results indicate that a separating bridge is essential for guaranteeing long-lived CS states.

**Figure 11:**
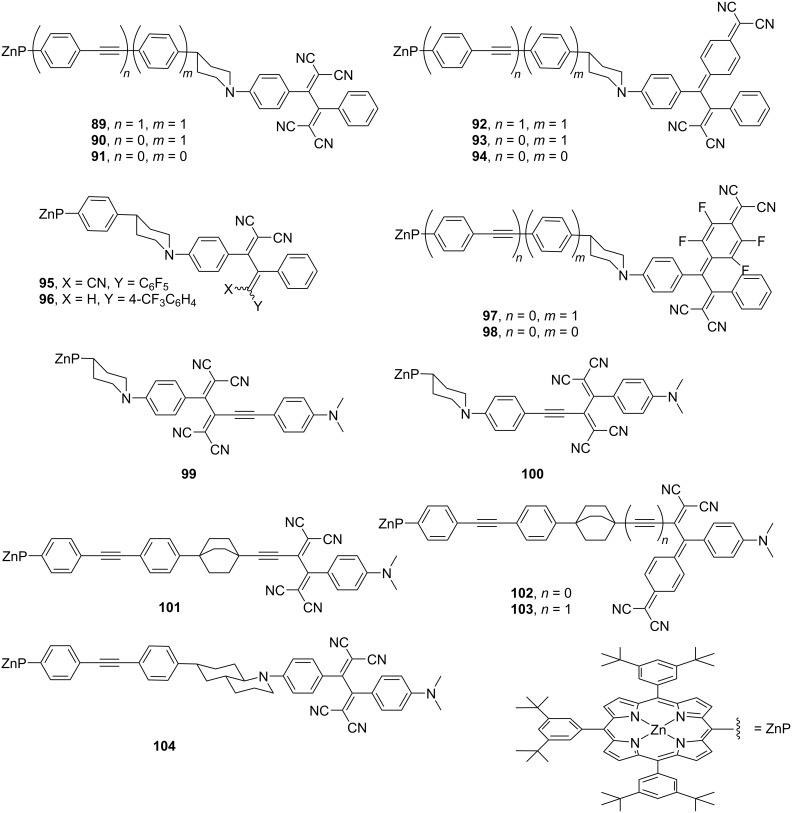
Structures of the porphyrin-based donor–acceptor conjugates (**89**–**104**).

Guldi et al. synthesized 16 distinct donor–acceptor conjugated molecules, denoted as **89**–**104** (Figure 11). These molecules comprised ZnP as an electron donor and push–pull chromophores with varying reduction potentials as electron acceptors interconnected by various rigid spacers [147–148]. Comprehensive analyses and characterizations of the photoinduced electron-transfer processes in these conjugates were conducted. In these molecular designs, the center-to-center distances of the electron donor and acceptor ranged from 13.9 Å to 25.1 Å. Marcus curves were obtained by measuring the charge-separation and charge-recombination lifetimes of these molecules by laser-flash photolysis (excitation wavelength = 420 nm) and the resulting electron-transfer rates were plotted. In the measurements in toluene, the lifetimes of the CS states and charge-recombination events differed significantly depending on the type of spacer and acceptor moiety, with the CS-state lifetimes ranging from 3 (for **98**) to 931 ps (for **103**) and the charge-recombination lifetimes ranging from 12 (for **98**) to 24,000 ps (for **101**). The obtained plot is consistent with the Marcus curves obtained using the semi-empirical approach, which includes the nuclear factor, particularly the electron-vibration coupling. This indicates that the consideration of quantum chemical vibrational effects is important in explaining the electron-transfer processes in these conjugates. For these molecules, the dynamics of the charge-recombination processes were found to be located in the Marcus inverted region. The total reorganization energies and electronic coupling matrix elements estimated from the fitting of the Marcus curve were determined to be 0.66–0.79 eV and 13.9–30.9 cm^−1^, respectively. Using a molecular design similar to that of **102**, the rectification property of a compound wherein TTF and DCNQ are linked by a rigid spacer has also been reported [149].

**Figure 12:**
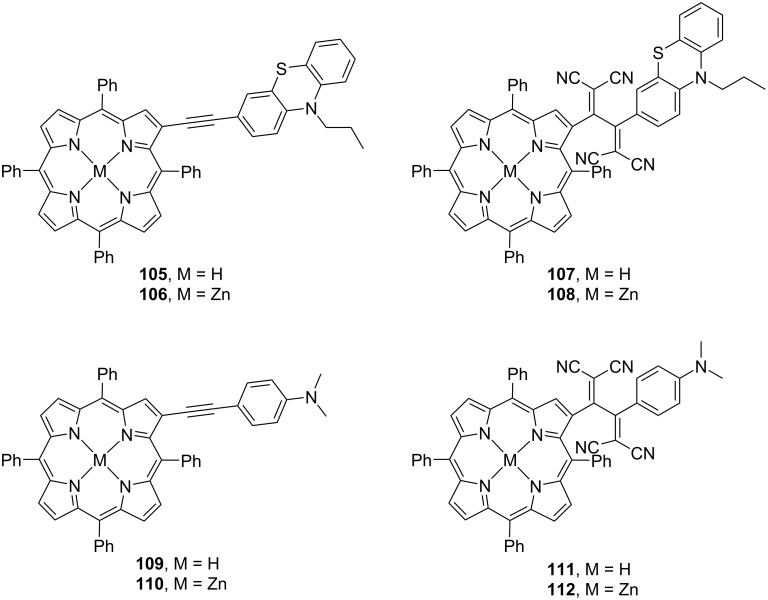
Structures of the porphyrin–PTZ or DMA conjugates **105**–**112**.

Misra et al. designed and synthesized a series of chromophore molecules **105**–**112** featuring porphyrin moieties and investigated their photophysical properties. They found that the introduction of the TCBD structures switched the role of porphyrins in photoevents (Figure 12) [150]. In molecules **105**, **106**, **109**, and **110** without the TCBD structure, when the metalloporphyrin moiety (MP) was subjected to Soret-band excitation (426 nm) in benzonitrile, the emergence of the S_2_ state was observed; the subsequent internal conversion of S_2_ into the S_1_ state was also observed, from which the CS state (MP^•−^–D^•+^) emerged. Here, since the triplet state ^3^MP* is energetically lower than the CS state, ^3^MP* can partially emerge at the intersystem crossing from the S_1_ state; a transition from the CS state to ^3^MP* is also possible. In contrast, for molecules **107**, **108**, **111**, and **112** bearing the TCBD structure, the S_2_ state emerges due to Soret-band excitation (426 nm) in benzonitrile. Further, after the transition from S_2_ to S_1_ by internal conversion, a CT state emerges: MP^δ+^–TCBD^δ−^–donor, where donor = phenothiazine (PTZ) or DMA. From this CT state, electron transfer occurs to form a CS state (MP^•+^–TCBD^•−^–donor), which returns to the ground state by charge recombination. These results indicate that MP functions as an electron acceptor in molecules without the TCBD structure, while the presence of the TCBD structure causes the porphyrin molecule to behave as an electron donor.

**Figure 13:**
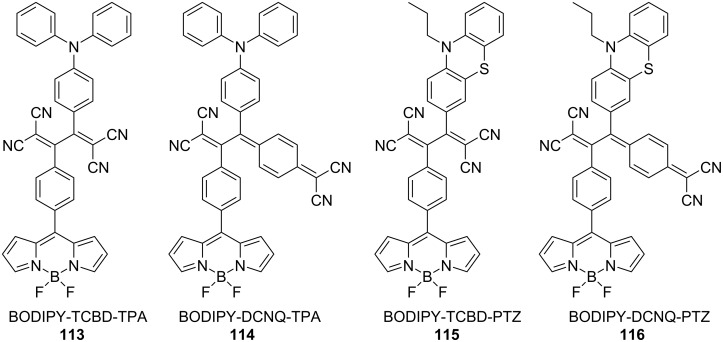
Structures of the BODIPY–Acceptor–TPA or PTZ conjugates **113**–**116**.

Misra et al. synthesized a series of TCBD and DCNQ derivatives with BF_2_-chelated BODIPYs and reported their photophysical properties [1,151]. Notably, for the CS state to emerge, it must have energy lower than the excited triplet state of BODIPY. Conjugates **113** and **114** have molecular structures wherein the TCBD or DCNQ moieties are inserted between the BODIPY and triphenylamine (TPA) moieties, as shown in Figure 13. The energy-level diagrams estimated from the Rehm–Weller equation suggested that at least two CS states were possible in the conjugates (BODIPY^•+^–acceptor^•−^–TPA and BODIPY–acceptor^•−^–TPA^•+^ (acceptor = TCBD or DCNQ)). In fact, when the transient absorption spectra of **113** and **114** were obtained using an excitation wavelength of 400 nm (the wavelength at which BODIPY is mainly excited), transient corresponding to the BODIPY^•+^–acceptor^•−^–TPA and BODIPY–acceptor^•−^–TPA^•+^ states were observed, highlighting ultrafast CS and charge-recombination events. In compounds **115** and **116** containing TCBD or DCNQ structures incorporated between BODIPY and PTZ, CS states, namely BODIPY–TCBD^•−^–PTZ^•+^ and BODIPY-DCNQ^•−^–PTZ^•+^, with a time constant of approximately 4 ps were observed after the formation of the excited singlet state of BODIPY by excitation at 511–512 nm in benzonitrile.

**Figure 14:**
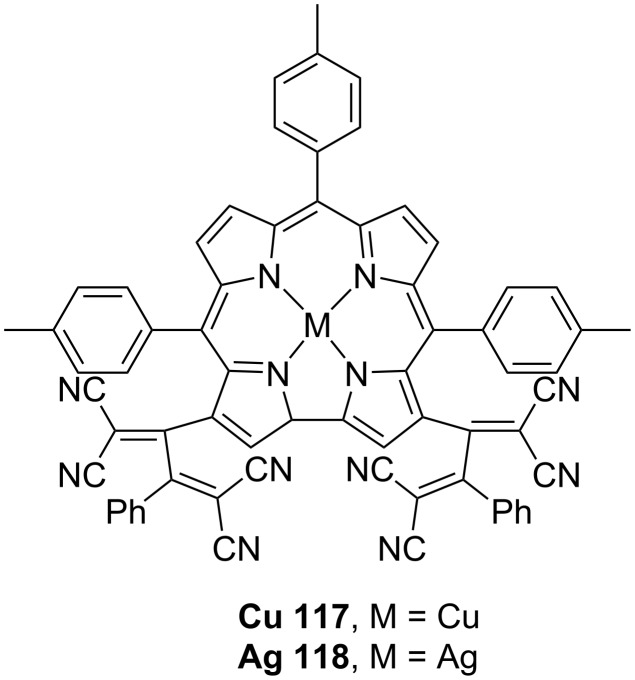
Structures of the corrole–TCBD conjugates **117** and **118**.

Sankar et al. designed and synthesized a conjugate consisting of TCBD coupled with a metal corrole to develop a system that can achieve an efficient population of the triplet states through the excited CT state [64]. TCBD derivatives containing copper or silver corrole complexes, denoted as **117** and **118**, respectively, were synthesized in a single step from their corresponding alkynyl precursors (Figure 14). The corresponding DCNQ derivatives were not successfully synthesized because of degradation during column purification. For these systems, the computationally calculated energy of the CT state (*E*_CT_) was lower than the excitation energies at the Soret (i.e., S_2_) and visible peak positions. Furthermore, the *E*_CT_ was higher than the triplet-state energies of the metal corroles. When **117** was subjected to the Soret-band (426 nm) excitation in benzonitrile, a short-lived, vibrationally hot S_1_ state emerged, along with a broad absorption peak at ≈592 nm, which is characteristic of the triplet state; the decay time constant of this state was 4.98 ns. Although the expected spectral features of the CT state were not observed in the transient absorption spectra because they were overwhelmed by the absorption band of the hot S_1_ state, the results suggested that the hot S1 state formed from the internal conversion of the initial S_2_ state promoted the formation of the CT state. Conversely, no transient absorption corresponding to the triplet state was observed when **117** was subjected to the visible band excitation (S_1_). In contrast, for **118**, a triplet state was observed for both S_2_ and S_1_ excitations, with time constants of 3.5 ns and 86 ps, respectively.

### Material applications

**Figure 15:**
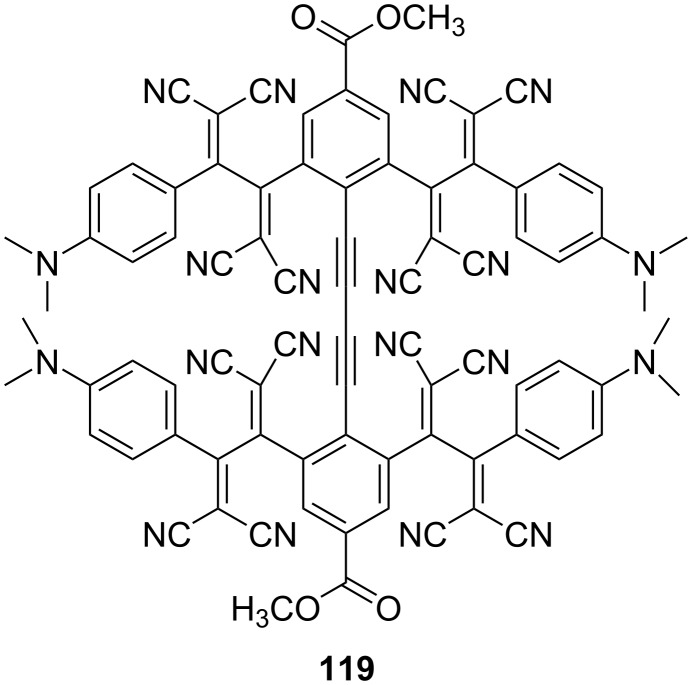
Structure of the dendritic TCBD **119**.

Due to their high molecular conversion efficiency, [2 + 2] CA–RE reactions have been employed to develop dendritic systems [55,63,152–155]. He et al. reported the synthesis of **119,** which contains multiple dendritic TCBD motifs that form porous molecular crystals (Figure 15) [156]. The rotational freedom of the covalent linkage within **119** is postulated to be substantially restricted due to its pronounced steric hindrance. This tenacious microporous crystalline structure exhibited robust thermal stability (up to 200 °C), as confirmed by powder X-ray diffraction studies. Gas-sorption studies were also performed on the solid samples post activation at 120 °C, revealing CO_2_ adsorption with pronounced hysteresis. This may be attributed to the presence of narrow, slit-like pores and voids, as seen in the crystal lattice of **119**. The Langmuir surface area of the compound was determined to be 317 m^2^/g. Furthermore, the compound exhibits easy fabrication of its thin films due to exceptional crystalline integrity when deposited on glass plates, highlighting its good reproducibility in solution-based processes. Li et al. analyzed the aggregation tendencies of push–pull chromophores involving TCBD or DCNQ moieties in the benzothiazole framework. Their findings revealed potential for the generation of hollow microstructures characterized by spherical or tubular morphologies [157].

**Figure 16:**
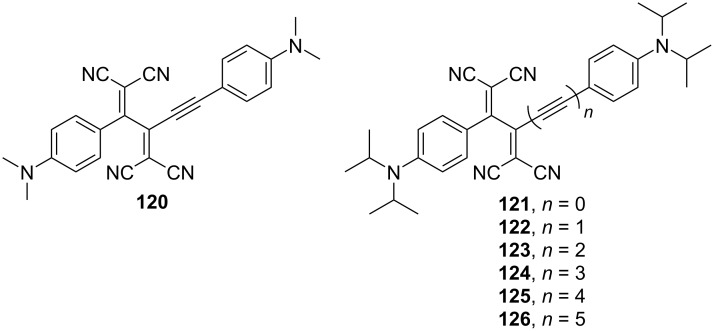
Structures of the TCBDs **120**–**126**.

TCBDs and related push–pull chromophores have attracted attention as organic nonlinear optical (NLO) materials because of their highly polarizable π-electron systems [158]. Thus far, second- and third-order NLO responses of several TCBD compounds have been evaluated [16,61,82,89,135,159–163]. Yang and Si theoretically calculated the second-order polarizability values that relate the second-order NLO responses of TCBD derivatives with different substituents and showed that the NLO properties can be tuned by changing the substituents [164]. Furthermore, Biaggio conducted a detailed study of the third-order NLO properties of TCBD derivatives – an overview is reported in a recent paper [165]. They experimentally determined the orientational average of the off-resonant third-order polarizability value (γ_rot_) of **120** (a benchmark compound) to be 12 ± 2 × 10^−48^ m^5^ V^−2^ (Figure 16). Furthermore, the γ_rot_ values of a series of compounds containing acetylene spacers of different lengths between the anilino group and TCBD moiety (**121**–**126**) have been determined, and a correlation between the length of the acetylene spacer and the γ_rot_ value was established: longer linking distances of the acetylene spacer result in larger the γ_rot_ values [160]. The appeal of **120** is its ability to undergo sublimation without decomposition and produce high-density, high-quality homogeneous molecular assemblies via molecular beam deposition [166]. Freude et al. fabricated a silicon–organic-hybrid slot waveguide by depositing **120** between two silicon ribs via molecular beam deposition and successfully demonstrated the all-optical demultiplexing of a 170.8 Gb s^−1^ telecommunication signal to 42.7 Gb s^−1^ [167].

**Figure 17:**
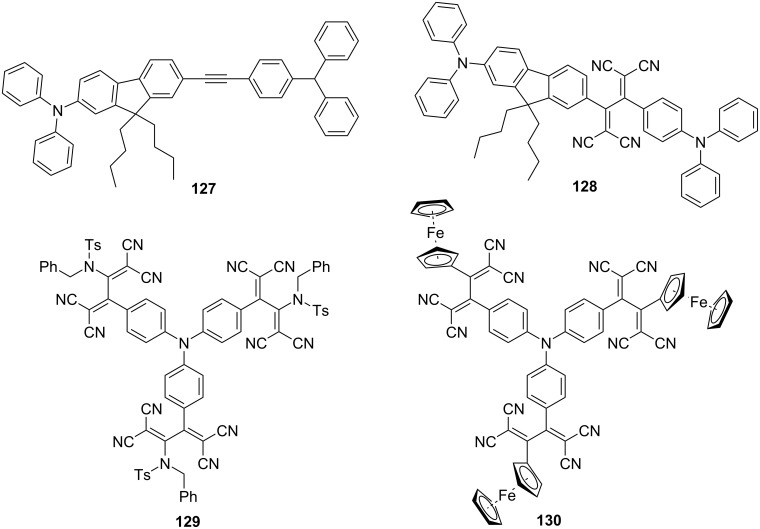
Structures of the precursor **127** and TCBDs **128**–**130**.

The two-photon absorption (2PA) properties of the TCBD derivatives have also been studied [135,168]. For example, the 2PA cross-section (σ_2_) of **128** measured by the Z-scan method was estimated to be 390 GM (2PA wavelength = 1,050 nm) (Figure 17) [135]. Although this value is smaller than that of the precursor (**127**; 540 GM; 2PA wavelength = 700 nm), **128** is expected to be used as a two-photon absorber in the NIR region. The branched TCBD derivatives **129** and **130** bearing the triarylamino core have also been synthesized and their 2PA properties have been evaluated. The σ_2_ values for **129** were 80 GM at 1,150 nm and 275 GM at 900 nm, and those of **130** were 90 GM at 1,150 nm and 350 GM at 900 nm, indicating that both compounds have relatively high 2PA cross-sections.

**Figure 18:**
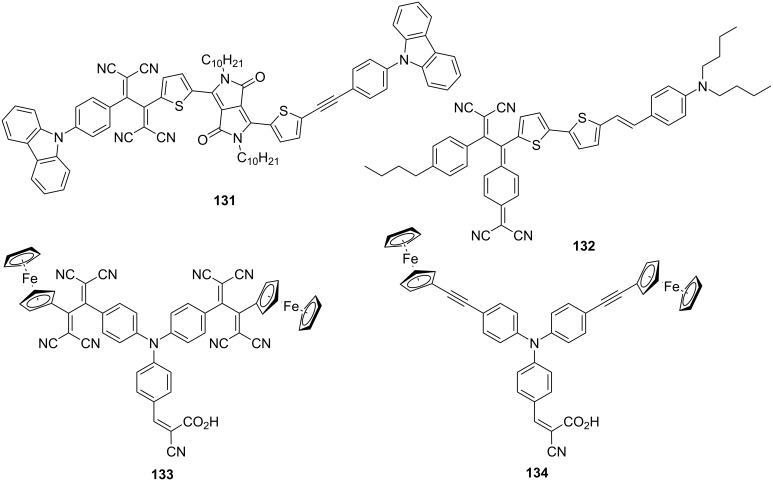
Structures of **131**–**134** utilized for BHJ OSCs.

TCBDs and their related push–pull chromophores have also been studied as candidate materials for applications in photovoltaic devices because of their exceptional redox properties. Notably, push–pull chromophores, such as TCBD and DCNQ, exhibit flexible highest occupied molecular orbital/lowest unoccupied molecular orbital levels that can be tuned by the appropriate selection of electron donor and acceptor [41,44–46,169–170]. To date, various bulk heterojunction organic solar cells (BHJ OSCs) based on TCBD derivatives have been developed and their photoelectric conversion properties have been evaluated. A comprehensive overview of the advances in this regard can be found in the work of Butenschön et al. [8]. While fullerene derivatives, such as [6,6]-phenyl-C_61_-butyric acid methyl ester ([60]PCBM) and [6,6]-phenyl-C_71_-butyric acid methyl ester ([70]PCBM) [171], have been widely used as electron-acceptor materials in BHJ OSCs, TCBD derivatives are considered as promising nonfullerene electron acceptors [26,172–176]. The power conversion efficiency of the BHJ OSCs fabricated using the TCBD derivative containing carbazole and diketopyrrolopyrrole structures, denoted as **131**, as the electron acceptor reached 7.19% (Figure 18) [173]. Some researchers have also focused on the introduction of electron-donating substituents into the TCBD structure and studied the use of TCBD as an electron donor in BHJ OSCs [24–25,177–181]. Praveen et al. fabricated BHJ OSCs with a 1:1 blending ratio of the DCNQ derivative **132** as an electron donor and [70]PCBM as an electron acceptor and reported that the power conversion efficiency reached 7.79% [25]. Dye-sensitized solar cells using **133**, a compound with two ferrocenyl TCBD structures introduced into the TPA structure, as a sensitizer have also been fabricated, achieving a power conversion efficiency of 4.96% [182]. Further, a power conversion efficiency was 3.65% was obtained when the precursor compound **134** was used. Therefore, the light-harvesting capability of the TCBD derivative is expected to contribute to the conversion efficiency.

## Conclusion

In summary, recent research advancements have revealed that [2 + 2] CA–RE reactions can not only yield nonplanar push–pull chromophores characterized by TCBD and DCNQ structures but also an array of π-electron compounds featuring attractive electronic and optical properties. These push–pull chromophores have been integrated into various molecules, including CPP, rotaxane, and fullerenes. Furthermore, studies have elucidated the linear and nonlinear optical characteristics of these compounds along with the dynamics of their excited states. Substantial progress has been made in the research on their utilization in photoelectric conversion and NLO materials. Future studies on these compounds should focus on improving their physical properties and functions to surpass those of the existing molecules.
